# The study of the role of purified anti-mouse CD193 (CCR3) antibody in allergic rhinitis mouse animal models

**DOI:** 10.1038/s41598-024-51679-3

**Published:** 2024-01-11

**Authors:** Youwei Bao, Zhaokun Wu, Xinhua Zhu, Jun Wu, Yinli Jiang, Ying Zhang, Yu Zhu, Zheng Liu, Yi Deng, Wenqiang Liu, Mengyi Wei, Weiming Luo, Yating Xiao

**Affiliations:** Department of Otolaryngology Head & Neck Surgery, the Second Affiliated Hospital of Nanchang University, Nanchang, 330006 China

**Keywords:** Asthma, Inflammatory diseases, Inflammatory diseases, Cytokines, Inflammation, Acute inflammation, Immunology, Diseases

## Abstract

The pathogenesis of allergic asthma is similar to that of allergic rhinitis, with inflammation cells producing and releasing inflammatory mediators and cytokines closely related to CCR3.Based on the theory of "one airway, one disease", the use of CCR3 monoclonal antibody may have a similar effect on allergic rhinitis. However, there are few studies on CCR3 monoclonal antibody in allergic rhinitis. Therefore, the aim of this study was to investigate the effective concentration of CCR3 monoclonal antibody, to compare the effects of different methods of administration, and to examine the lung condition of allergic mice to investigate whether antibody treatment protects the lungs. In this study, we constructed a mouse model of allergic rhinitis and intraperitoneally injected different doses of CCR3 monoclonal antibody (5, 10, and 20 uL/mg) to observe its therapeutic effect: observing changes in tissue morphology of nasal mucosa, infiltration of inflammation, and using ELISA to detect changes in relevant inflammatory mediators and cytokines, studying the role of CCR3 mAb in inhibiting CCR3-related actions on the nasal mucosa of allergic rhinitis mice. Furthermore, In addition, the therapeutic effects of intraperitoneal injection (i.p.) and intranasal administration (i.n.) were studied on the basis of effective concentrations.

## Introduction

Allergic rhinitis (AR) is a type I allergic reaction in the nose triggered by exposure to specific allergens in individuals with atopy, mediated by IgE and involving various cytokines and inflammatory mediators. The inflammation in AR primarily involves eosinophils, Th2 cells, and mast cells, which all express CCR3 on their surfaces^1–4^.

Previous studies have reported the important role of the CCR3 pathway in the activation of eosinophils in vivo and have also demonstrated increased CCR3 expression in allergic airway diseases. The pathogenesis of AR is closely related to CCR3 chemokine receptor 3 (CCR3). In this study, we used RNAi to silence CCR3 and found that it could inhibit the proliferation and promote apoptosis of mouse eosinophils. Deletion of the CCR3 gene in mice reduced the number of invasive eosinophils and the severity of inflammation in AR mice by downregulating the expression of EPO, ECP, MBP, IL-4, and IgE.

CCR3 monoclonal antibody (CCR3 mAb)^5–9^ is a highly uniform antibody produced by a single B cell clone that targets specifically to the CCR3 antigen epitope^10,11^. It is usually generated using hybridoma technology and can specifically block the downstream effects of CCR3 on cells. Therefore, using CCR3 mAb can inhibit the actions of inflammatory cells in an allergic state and reduce the amplification of allergic reactions. The CCR3 mAb used in this study was produced by BioLegend (AB_2715914, BioLegend Cat. No. 144502)^12,13^, which had previously been used for flow cytometry detection but was applied for therapeutic use in this experiment to explore its efficacy^14–17^.

## Results

### Statistical analysis of behavioral physiology in each group of mice

**Figure 1:**
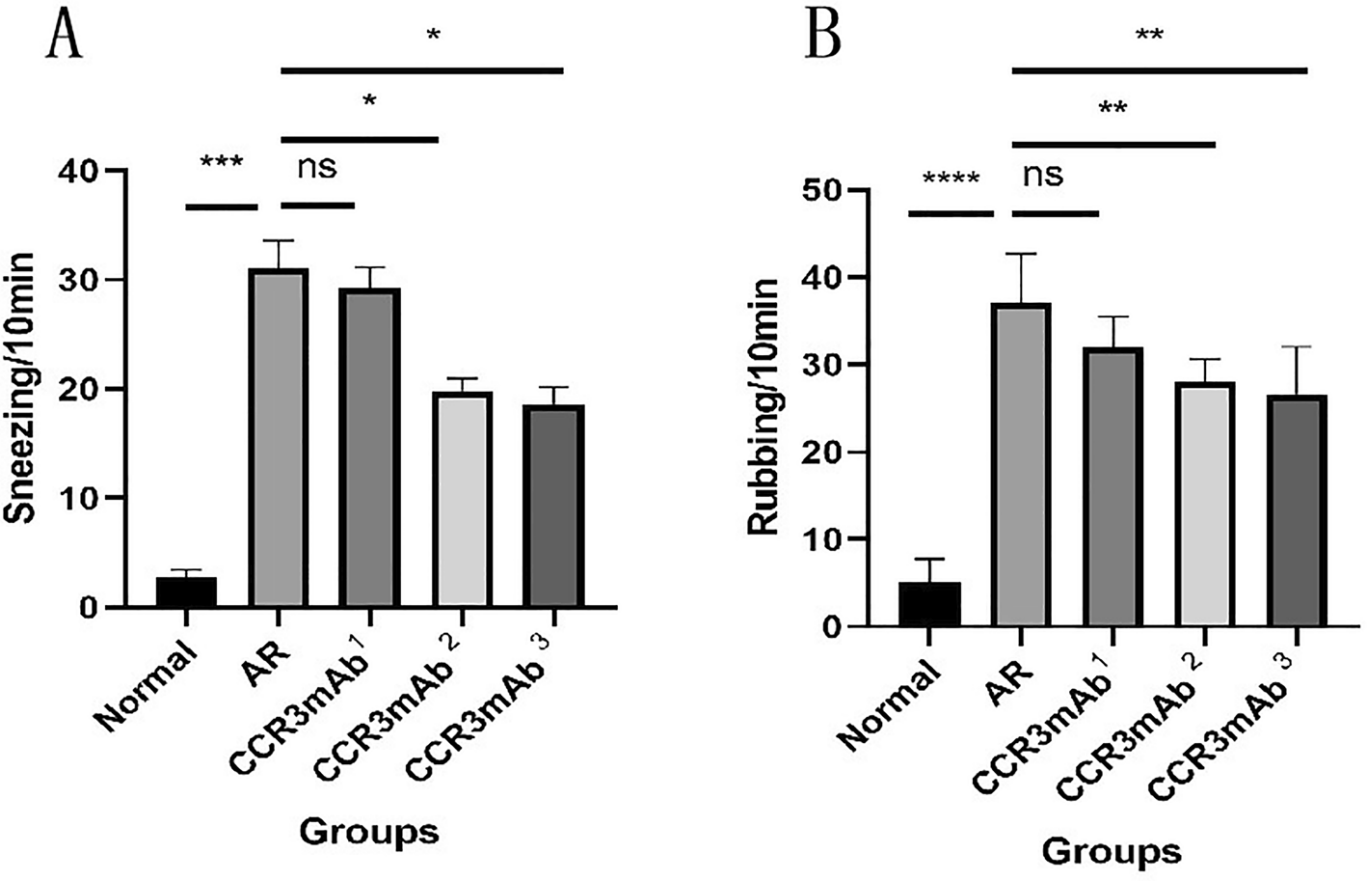
Gradient concentration administration: (**A**) Sneezing count after nasal challenge in mice; (**B**) Scratching nose count after nasal challenge in mice.

After the final mouse nasal OVA challenge, the number of sneezing and scratching nose counts within 10 min were counted. Through behavioral observation (Fig. 1A,B), the average scratching nose time for normal control mice was 5.20 ± 1.16 s/time, and the average sneezing time was 2.80 ± 0.66 s/time; for allergic rhinitis group (AR group), significant symptoms of scratching nose and sneezing were observed, with an average of 37.20 ± 2.46 s for a scratching nose action and 31.00 ± 2.63 s for a sneezing action; in the CCR3mAb1 group, the average scratching nose time was 32.00 ± 1.58 s/time, and the average sneezing time was 29.20 ± 1.93 s/time; in the CCR3mAb2 group, the average scratching nose time was 28.00 ± 1.18 s/time, and the average sneezing time was 19.80 ± 1.16 s/time; in the CCR3mAb3 group, the average scratching nose time was 26.60 ± 2.42 s/time, and the average sneezing time was 18.60 ± 1.57 s/time. Based on the above data, it can be found that the CCR3mAb23 i.p. group also had obvious scratching nose and sneezing, but compared to the AR group, it was significantly alleviated, and there was a statistical significance (P < 0.05), but it was still higher than the normal control group, especially in the allergic rhinitis group (all P˂0.05). However, there was no significant difference in the number of sneezing and scratching nose counts between the CCR3mAb1 i.p. group and AR group (Fig. 1). The behavioral observation and analysis of mice showed that using CCR3mAb could improve symptoms of allergic rhinitis^18–20^.

**Figure 2:**
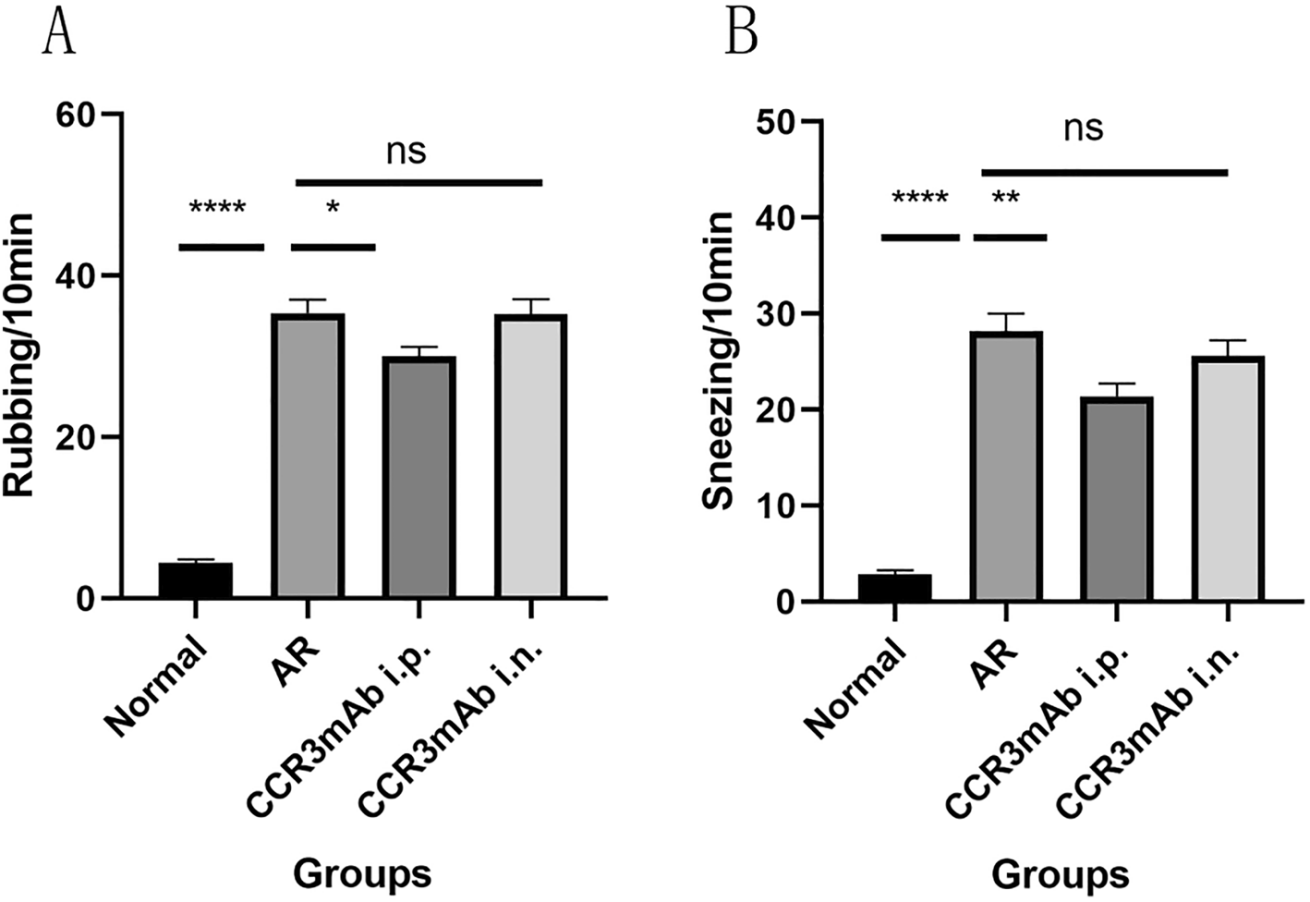
Different modes of administration: (**A**) Sneezing count after nasal challenge in mice; (**B**) Scratching nose count after nasal challenge in mice.

Through behavioral observation (Fig. 2A,B), based on the above data, it can be found that the CCR3mAb i.p. group also had obvious scratching nose and sneezing, but compared to the AR group, it was alleviated, and there was a statistical significance (P < 0.05), but it was still higher than the normal control group, especially in the allergic rhinitis group (all P < 0.05). However, there was no significant difference in the number of sneezing and scratching nose counts between the CCR3mAb i.n. group and AR group. The behavioral observation and analysis of mice showed that using CCR3mAb intraperitoneal injection could improve symptoms of allergic rhinitis, while topical application had no difference.

### Detection of mouse nasal mucosa (HE X400)

**Figure 3:**
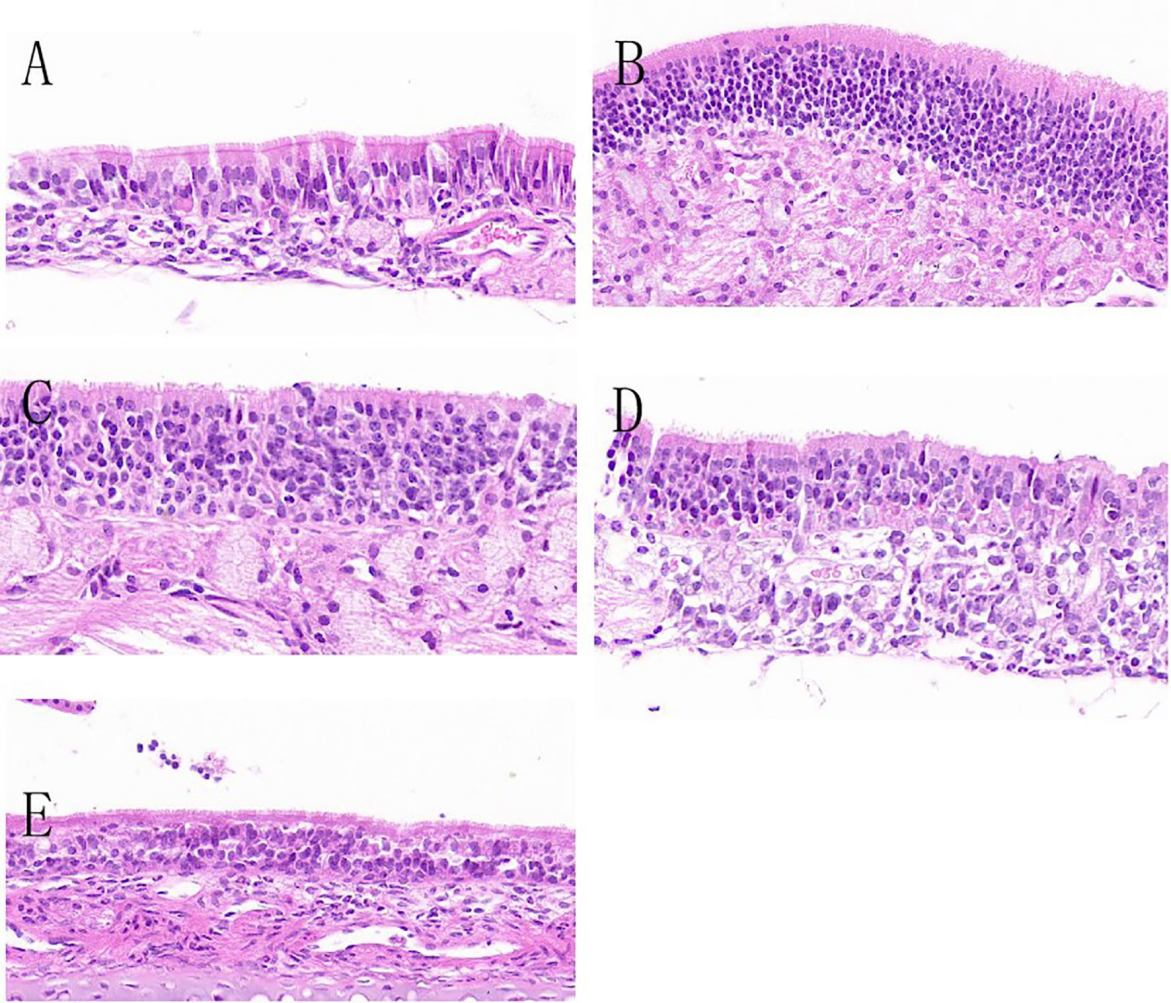
Gradient concentration administration: HE staining results of nasal mucosa. (**A**) Normal control mice; (**B**) allergic rhinitis group mice; (**C**) mice injected with CCR3mAb^1^; (**D**) mice injected with CCR3mAb^2^; (**E**) mice injected with CCR3mAb^3^.

The results showed that in the normal control group, the nasal mucosa tissue structure was clear, intact, smooth surface, and no significant swelling of the mucosa and submucosa. There was only a small amount of inflammatory cells infiltrating the tissue, with no significant dilation of glands in the intrinsic layer of the mucous membrane, and clear cilia could be observed with well-organized arrangement (Fig. 3A).

In the allergic rhinitis group, the nasal mucosa structure was unclear, and there was a large number of inflammatory cells infiltrating the tissue. The mucosa and submucosa were significantly swollen, with a blurred boundary, and cilia were destroyed, disordered, and shed. Some epithelial cells were damaged and detached (Fig. 3B). Compared to the normal control group, it showed significant signs of inflammation. However, when comparing the AR group with the CCR3mAb^1^ i.p. group, the arrangement of cilia in the mucosa was slightly more organized, and there was no significant difference in terms of inflammation infiltration (Fig. 3B,C). In the CCR3mAb^2^ i.p. group, the overall structure of the nasal mucosa was still clear. The shedding of upper cilia was less severe than in the AR group. The boundary between the mucosa and submucosa could still be differentiated, and the infiltration of inflammatory cells in the mucosa and submucosa was significantly reduced compared to the allergic rhinitis group (Fig. 3D). In the CCR3mAb^3^ i.p. group, the overall structure of the nasal mucosa was more distinct. The structure of upper cilia partially recovered, and the mucosa and submucosa returned to their normal structure. The infiltration of inflammatory cells in the mucosa and submucosa was also significantly reduced compared to the allergic rhinitis group (Fig. 3E).

After applying CCR3mAb intervention to allergic rhinitis mice, there was a suppression effect on inflammation of the nasal mucosa of allergic rhinitis mice. This not only alleviated the infiltration of inflammatory cells but also allowed for a certain degree of recovery of swollen mucosa and disordered cilia. With increasing concentrations of CCR3mAb, the therapeutic effect became more pronounced, which is considered to be due to the fact that antibody concentration has not yet reached its maximum effect^21,22^.

**Figure 4:**
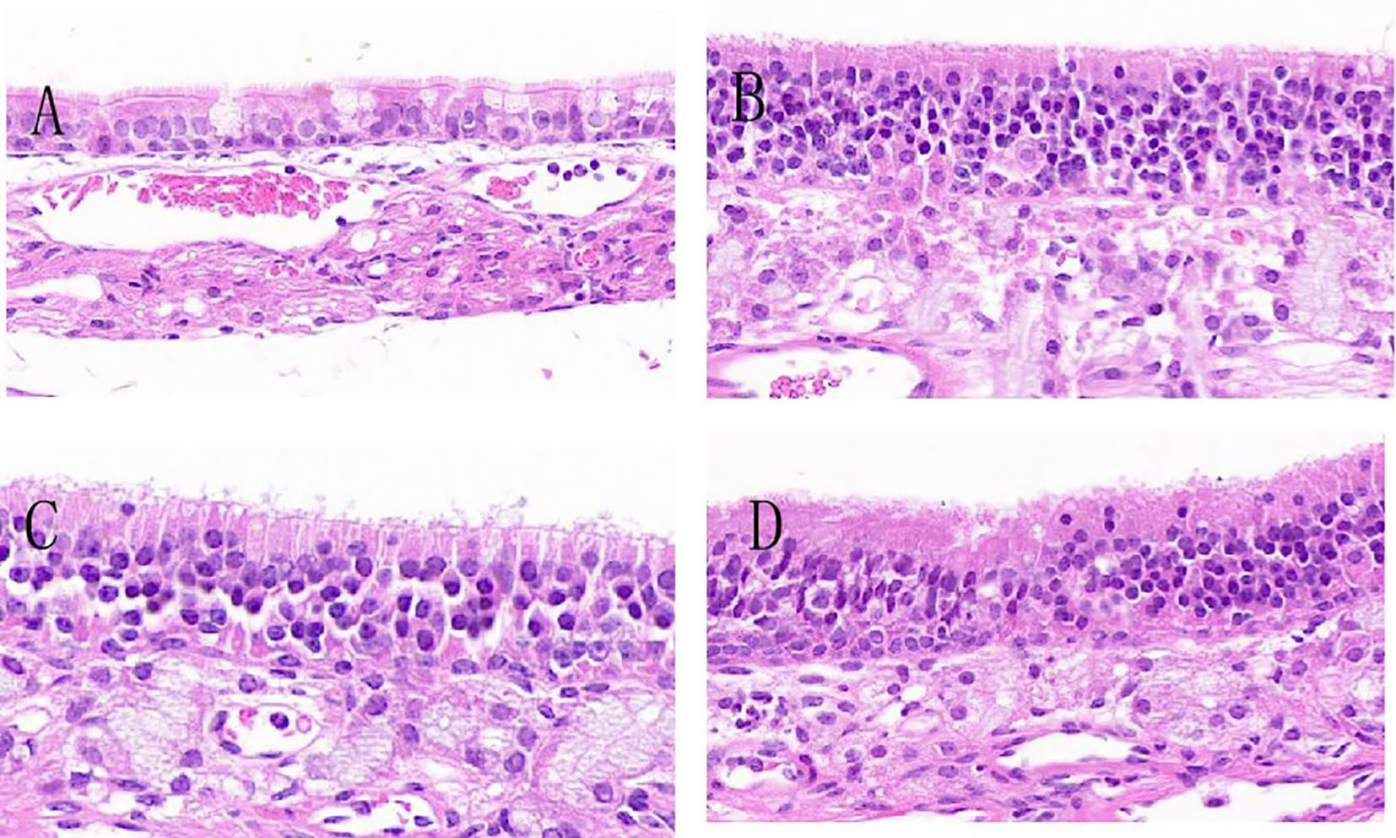
Different modes of administration: HE staining results of nasal mucosa. (**A**) Mice in the normal control group; (**B**) Mice in the allergic rhinitis group; (**C**) Mice injected with CCR3mAb intraperitoneally; (**D**) Mice administered with CCR3mAb intranasal administration

The results showed that in the allergic rhinitis group, the nasal mucosa structure was unclear, and there was a large number of inflammatory cells infiltrating the tissue. The mucosa and submucosa were significantly swollen, with a blurred boundary, and cilia were destroyed, disordered, and shed. Some epithelial cells were damaged and detached, showing significant signs of inflammation compared to the normal control group (Fig. 4A,B). In the CCR3mAb i.p. group, the overall structure of the nasal mucosa was still clear. The shedding of upper cilia was less severe than in the AR group. The boundary between the mucosa and submucosa could still be differentiated, and the infiltration of inflammatory cells in the mucosa and submucosa was not significantly different from that in the allergic rhinitis group (Fig. 4C). However, when comparing the CCR3mAb i.p. group with the AR group, there was disordered arrangement of cilia and no significant difference in terms of inflammation infiltration (Fig. 4B,D).

After applying CCR3mAb intervention through intraperitoneal injection to allergic rhinitis mice, there was a suppression effect on inflammation of the nasal mucosa of allergic rhinitis mice. This not only alleviated the infiltration of inflammatory cells but also allowed for a certain degree of recovery of swollen mucosa and disordered cilia. However, when comparing the CCR3mAb i.p. group with the allergic rhinitis group, there was no significant difference in this regard (Fig. 4D).

### Number of eosinophils in nasal mucosa (HE X400)

For each mouse in each group, take 5 HE-stained sections at random from the nasal mucosa of each mouse under a microscope with a 400 × field of view. Calculate the average value. Count the number of eosinophils in the field of view, only take one side from top and bottom, and only one side from left and right^23^.

**Figure 5:**
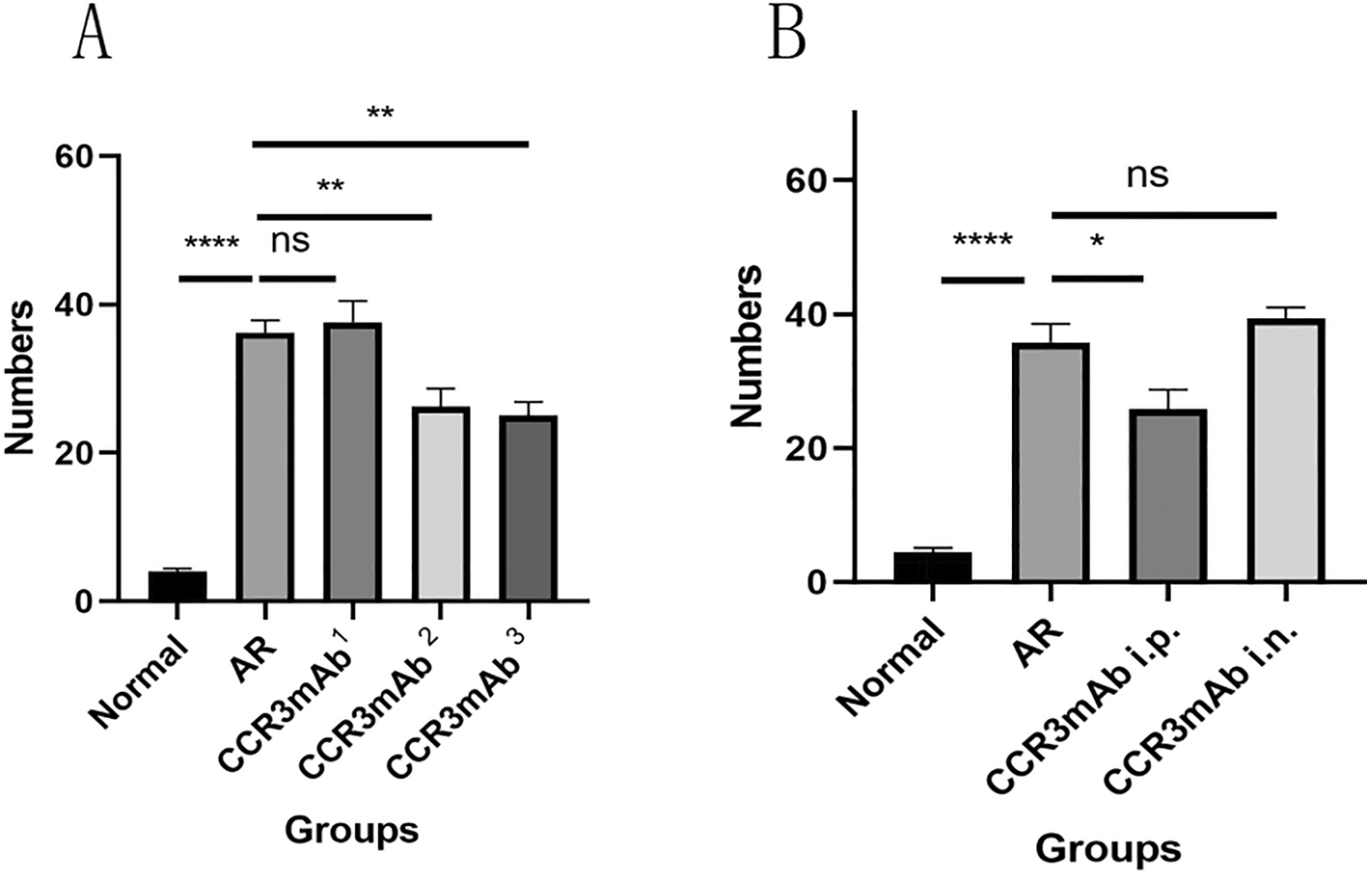
(**A**) Gradient concentration administration: number of eosinophils under 400-fold magnification by HE staining. (**B**) Different drug administration methods: HE staining at 400 × magnification for the number of eosinophils.

After administering different concentrations of drugs, we found that except for the CCR3mAb^1^ group, there was no significant statistical difference compared to the allergic rhinitis group. The CCR3mAb^2^ and CCR3mAb^3^ groups had a downward trend and showed significant statistical differences compared to the allergic rhinitis group (Fig. 5A).

After administering different drug administration methods, we found that the CCR3mAb i.p. group had a downward trend and showed significant statistical differences compared to the allergic rhinitis group. The CCR3mAb i.n. group showed no significant statistical difference compared to the allergic rhinitis group (Fig. 5B).

### Measurement of nasal mucosa thickness (HE X400)

The mouse nasal mucosa is divided into three layers: basal membrane cells, epithelial cell layer, and ciliary cells. In allergic rhinitis mice, the ciliary layer exhibits ciliary shedding, the epithelial cell layer thickens, the basal membrane cell layer shows disorganization, and the number of glands increases significantly. To quantify the allergic state, we are now counting the thickness data of the nasal mucosa to gain a more intuitive understanding of the allergic state. We scanned the HE-stained sections under an electron microscope, placing each slide at the 400 × field of view in each group, and randomly selecting 5 measurement data for recording.

**Figure 6:**
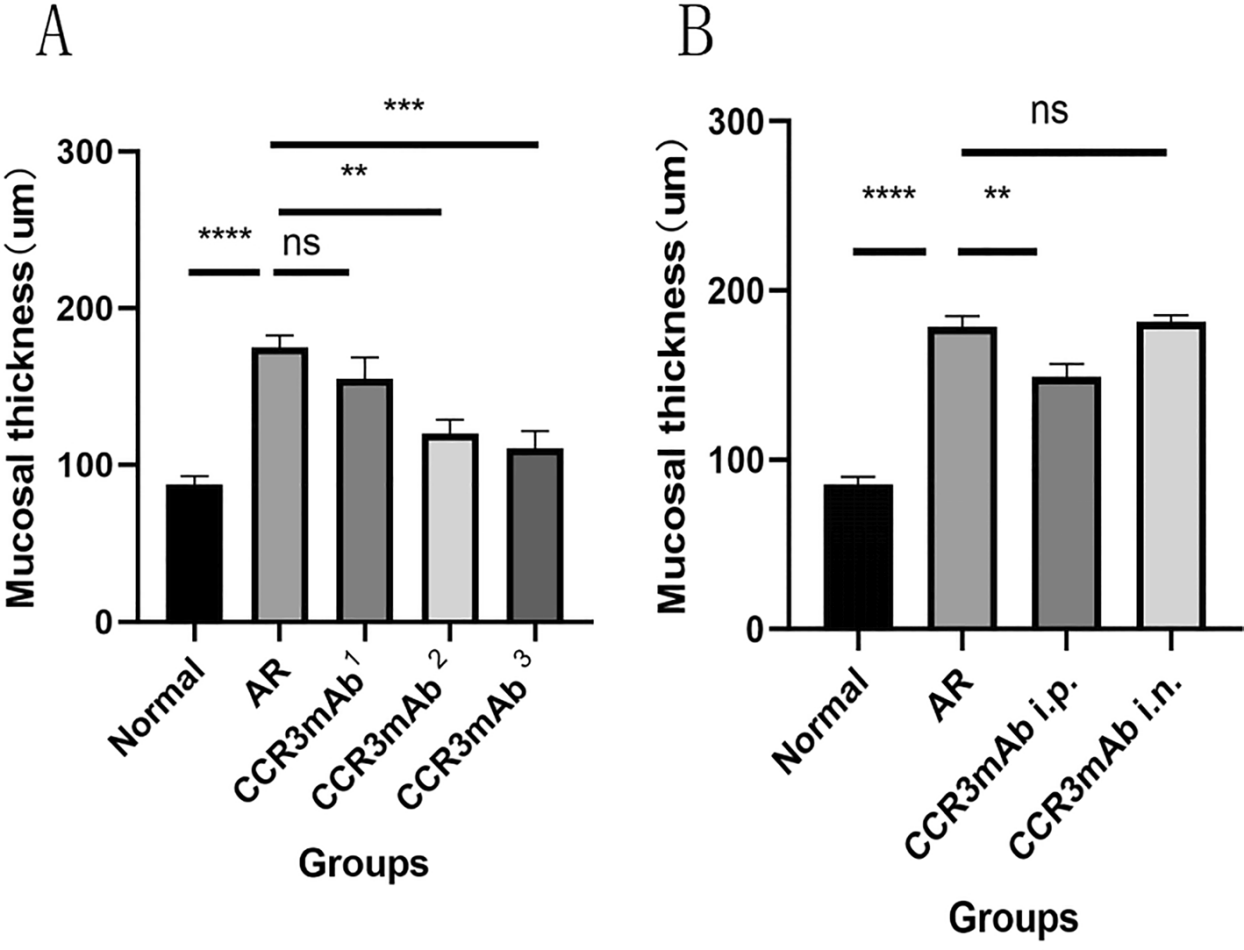
(**A**) Gradient concentration administration: number of eosinophils under HE staining at 400 × magnification. (**B**) Different administration methods: HE staining at 400 × magnification for thickness of mucosa.

Allergic rhinitis group mice have thicker mucosal thickness than normal group mice, with no significant statistical difference between CCR3mAb^1^ group and allergic rhinitis group. CCR3mAb^2^ and CCR3mAb^3^ groups have thicker mucosal thickness than allergic rhinitis group and show statistically significant differences (Fig. 6A).This indicates that intraperitoneal injection has therapeutic effects, while topical administration is ineffective (Fig. 6B).

### Mouse lung tissue examination (HE X400)

HE staining was used to observe and compare the pathological morphological changes and inflammatory cell infiltration of the mucous membrane in each group of mice. The morphology of mouse mucosa tissue and inflammatory cell infiltration were observed under a high-power microscope (400 ×) field.

**Figure 7:**
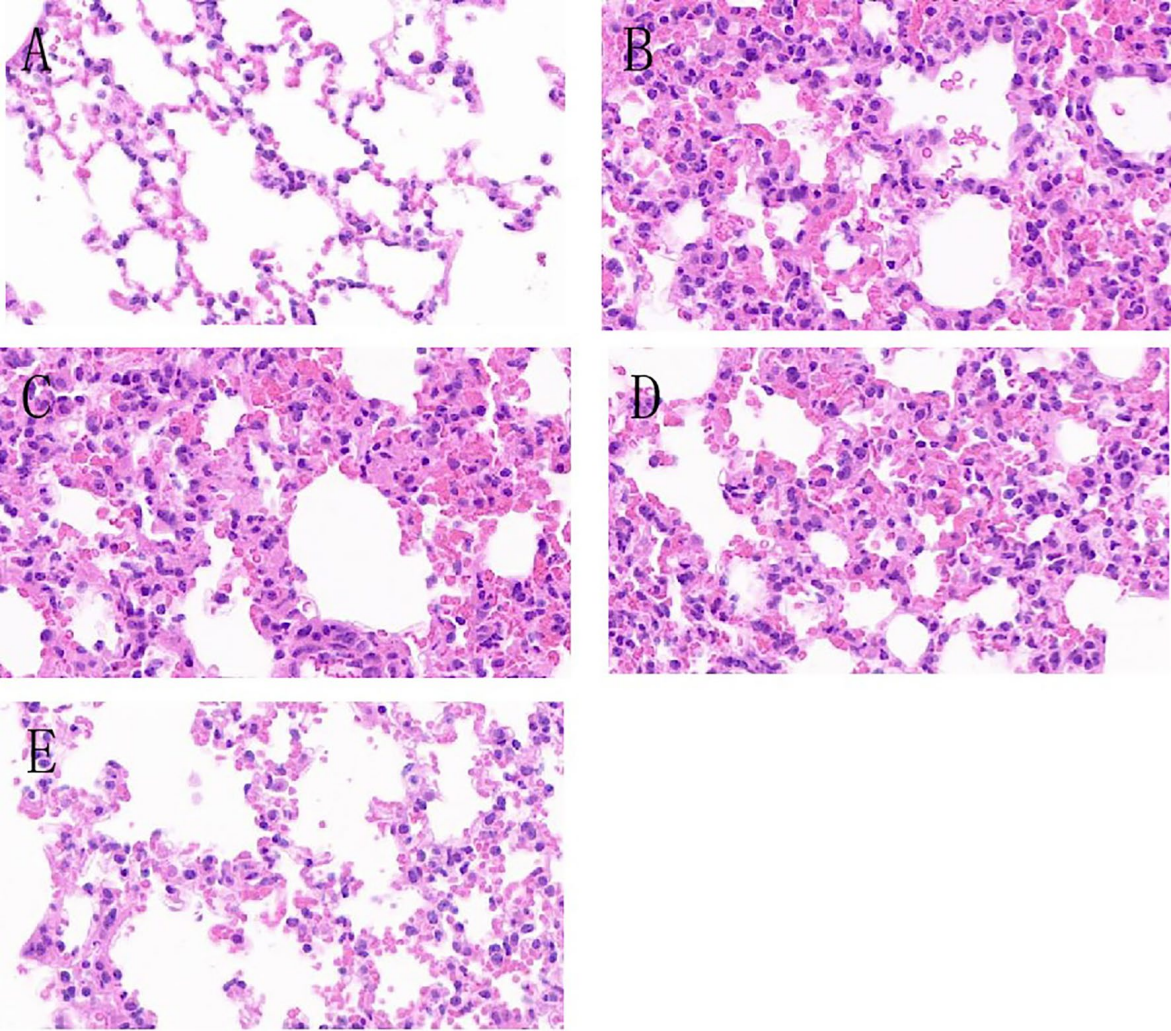
Gradient concentration administration: HE staining results of mouse nasal mucosa. (**A**) Normal control group mice; (**B**) Allergic rhinitis group mice; (**C**) CCR3mAb^1^ injection group mice; (**D**) CCR3mAb^2^ injection group mice; (**E**) CCR3mAb^3^ injection group mice.

After administration with gradient concentrations, normal group (NC) had clear organization of lung tissue, filled alveoli, no obvious damage, and no significant inflammation cells infiltration in the interstitial spaces of the alveolar tissues, nor obvious congestion or abnormal changes (Fig. 7A). Allergic group (AR) showed normal alveolar tissue not visible, numerous inflammation cells infiltrating between alveoli, and marked congestion (Fig. 7B). CCR3mAb^1^ group and CCR3mAb^2^ group were compared with AR group, both showing significant inflammation but no obvious abnormalities (Fig. 7C,D). CCR3mAb^3^ group showed significant inflammation compared with allergic AR group, alveolar tissue still visible but with marked inflammation and inflammation cells infiltrating in the interstitial spaces of alveoli, with congestion reduced. These results indicate that only CCR3mAb^3^ group has protective effects on the lung tissue of allergic mice, while the other two treatment groups have no significant differences from the allergic group (Fig. 7E).

**Figure 8:**
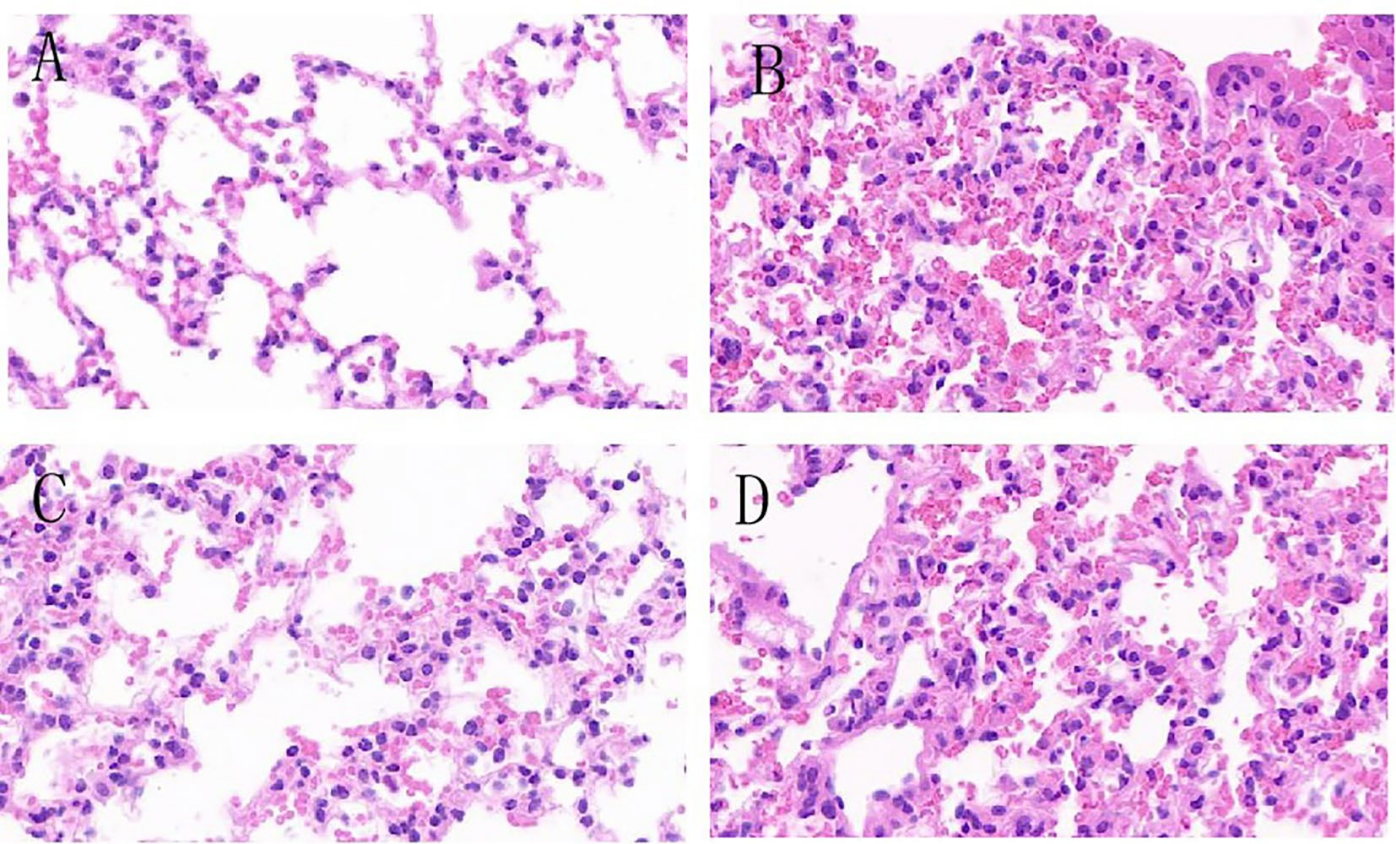
HE staining results of different administration methods on mouse nasal mucosa: (**A**) normal control group mouse; (**B**) allergic rhinitis group mouse; (**C**) CCR3mAb i.p. intraperitoneal injection group mouse; (**D**) CCR3mAb i.n. intranasal administration group mouse.

After different administration methods, normal group (NC) had clear organization of lung tissue, filled alveoli, no obvious damage, and no significant inflammation cells infiltration in the interstitial spaces of the alveolar tissues, nor obvious congestion or abnormal changes (Fig. 8A). Allergic group (AR) showed normal alveolar tissue not visible, numerous inflammation cells infiltrating between alveoli, and marked congestion (Fig. 8B). CCR3mAb i.p. group was compared with allergic AR group, both showing significant inflammation but no obvious abnormalities (Fig. 8C). CCR3mAb i.n. group was compared with AR group, both showing significant inflammation but no obvious abnormalities (Fig. 8D).

These results indicate that only CCR3mAb i.p. group has protective effects on the lung tissue of allergic mice, while CCR3mAb i.n. treatment group has no significant differences from the allergic group.

### ELISA detection results of cytokine levels in the peripheral blood tissue of mice

After collecting peripheral blood, the samples were left at 4 °C for a day and then centrifuged at 3000 rpm for 10 min. The supernatant was used to detect cytokines. According to the manufacturer's instructions, an immune analysis kit was used to detect serum IFN-γ, IL-2, IL-4, IL-5, and IL-13 levels using enzyme-linked immunosorbent assay (ELISA). IL-2 and IFN-γ are Th1 cell-related inflammatory cytokines, while IL-4, IL-5, and IL-13 are Th2 cell cytokines. These two types of indicators can also reflect the severity of inflammation. In this experiment, ELISA was also used to detect cytokines in mice's peripheral blood.

In the gradient concentration experiment, the results showed that the expression levels of Th1 cell cytokines (IL-2 and IFN-γ) in allergic rhinitis group were significantly lower than those in the blank control group and the CCR3mAb^23^ intraperitoneal injection group, with statistical significance. There was no significant difference compared to the CCR3mAb^1^ intraperitoneal injection group. For Th2 cell cytokines (IL-4, IL-5, and IL-13), except for no significant difference compared to the CCR3mAb^1^ group, they showed a downward trend compared to the normal group and the CCR3mAb^2,3^ group.

**Figure 9:**
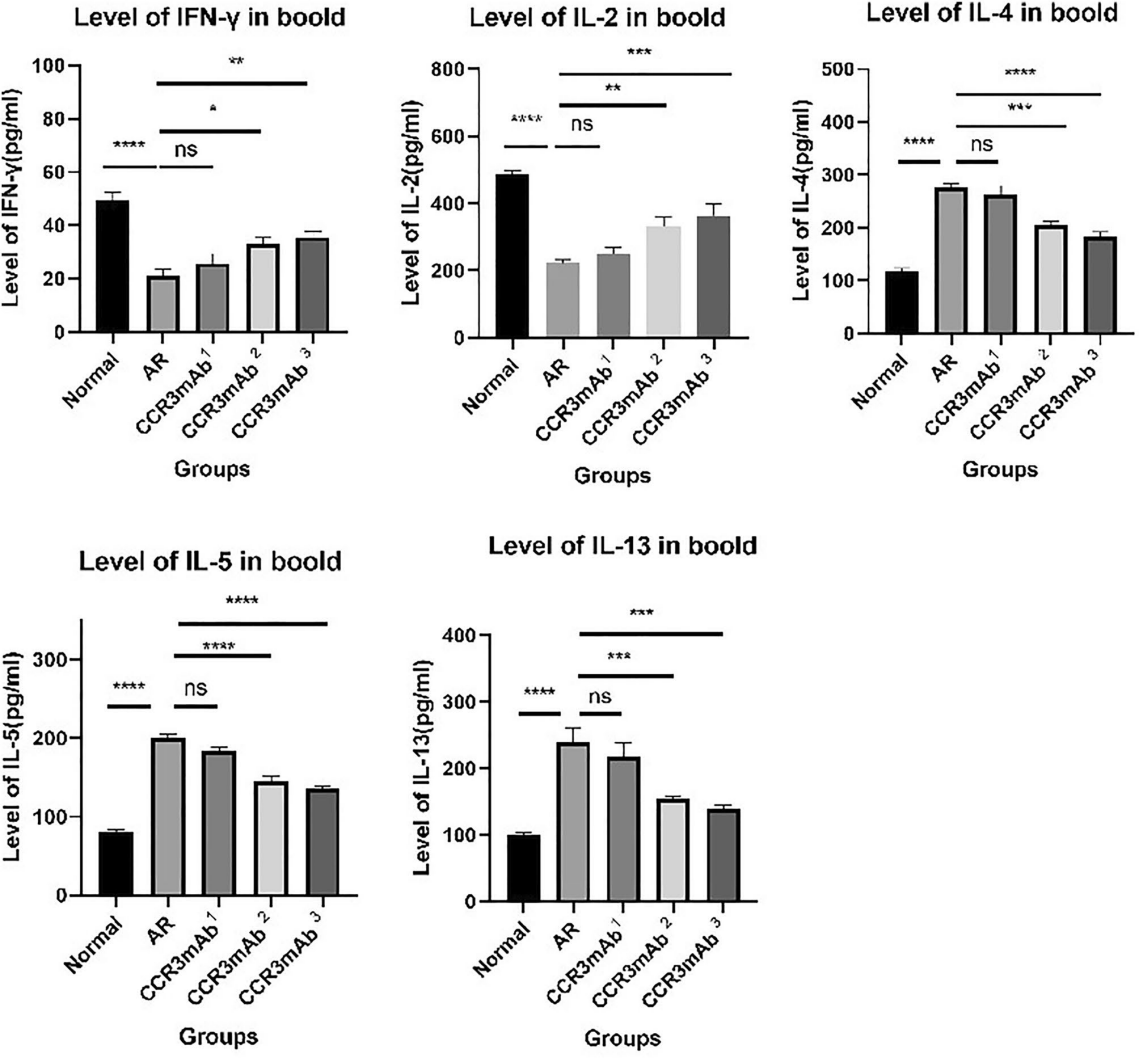
Gradient concentration administration: ELISA detection results of cytokine levels in the peripheral blood tissue of mice. *Indicates a significant difference compared with AR (P˂0.05); **Indicates a significant difference compared with AR (P˂0.01); ***Indicates a significant difference compared with AR (P˂0.001); ****Indicates a significant difference compared with AR (P˂0.0001).

Compared to allergic rhinitis mice, CCR3mAb^1^ group had no significant differences in Th1 and Th2 cytokines. Considering previous data, this may be due to insufficient drug concentration leading to insufficient therapeutic effect. Compared to allergic rhinitis mice, CCR3mAb^2^ and CCR3mAb^3^ groups had increased trends in Th1 cytokines (IL-2 and IFN-γ), with statistical significance; and decreased trends in Th2 cytokines (IL-4, IL-5, and IL-13) (Fig. 9).

**Figure 10:**
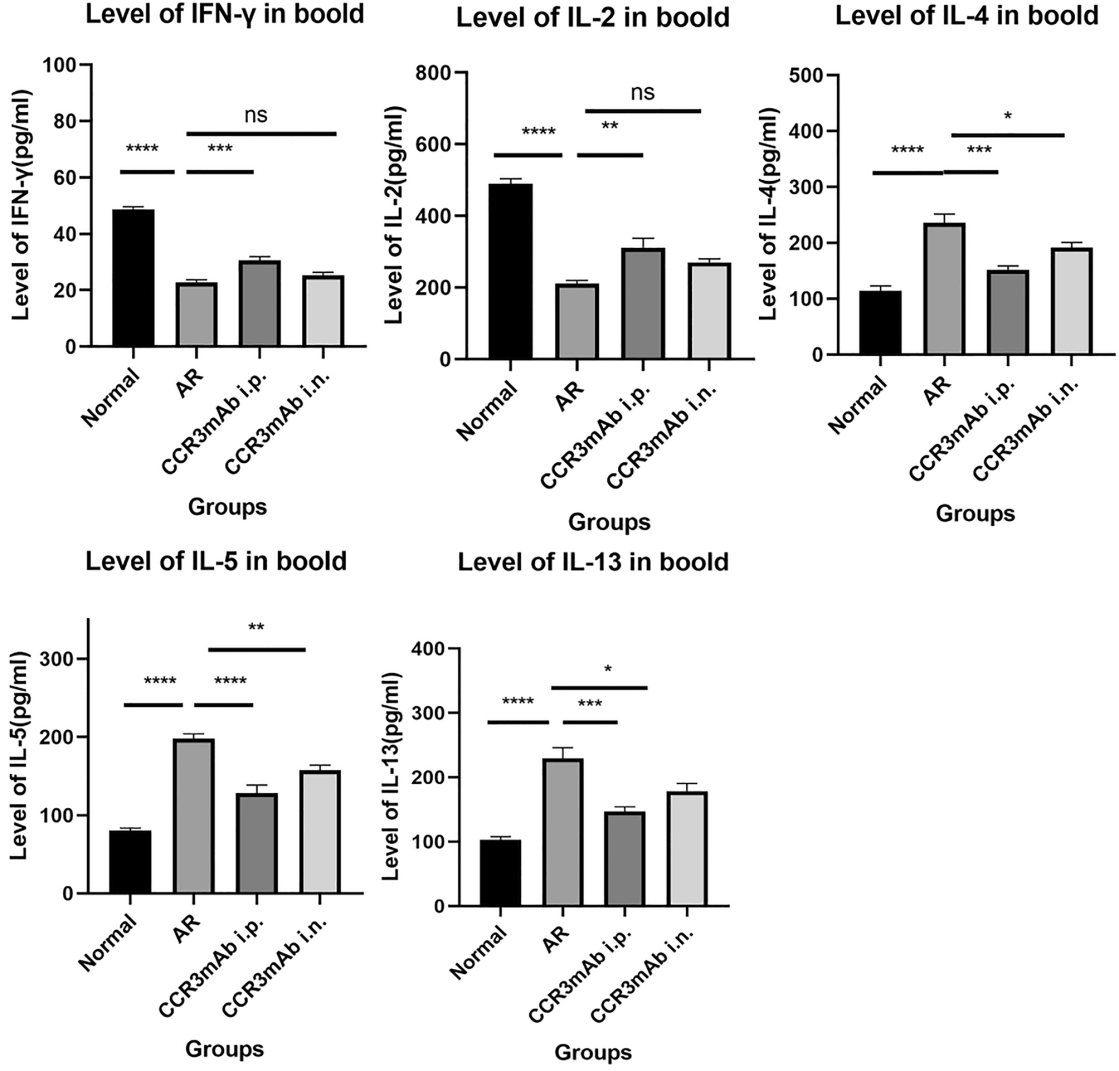
Different modes of administration: ELISA detection results of cytokine levels in the peripheral blood tissue of mice. *Indicates a significant difference compared with AR (P˂0.05); **Indicates a significant difference compared with AR (P˂0.01); ***Indicates a significant difference compared with AR (P˂0.001); ****Indicates a significant difference compared with AR (P˂0.0001).

After different administrations, CCR3mAb i.p. group had increased trends in Th1 cytokines (IFN-γ and IL-2) compared to allergic rhinitis mice, with statistical significance; and decreased trends in Th2 cytokines (IL-4, IL-5, and IL-13). However, CCR3mAb i.n. group had no significant difference in Th1 cytokines and showed a downward trend in Th2 cytokines compared to allergic rhinitis mice. In addition, based on these results, it is possible to roughly see the CCR3mAb intraperitoneal injection group. This proves that the use of CCR3mAb has a significant inhibitory effect on the release of inflammatory mediators such as IL-2 and IFN-γ byTh2 cells in allergic mice (Fig. 10).

All results demonstrate that the use of CCR3mAb has a significant relieving effect on respiratory allergy in mice. The mechanism is that CCR3mAb binds to the Fc domain of CCR3 expressed on the surface of mast cells, eosinophils, and other cells via its crystallizable region (Fc), thus blocking the Eotaxin/CCR3 pathway. Subsequently, it inhibits a series of activities downstream of mast cells and eosinophils (differentiation maturation, chemotaxis recruitment, cytokine and inflammatory mediator release and degranulation), thereby achieving the goal of relieving respiratory allergy.

### Changes in the net body weight increase of experimental mice

**Figure 11:**
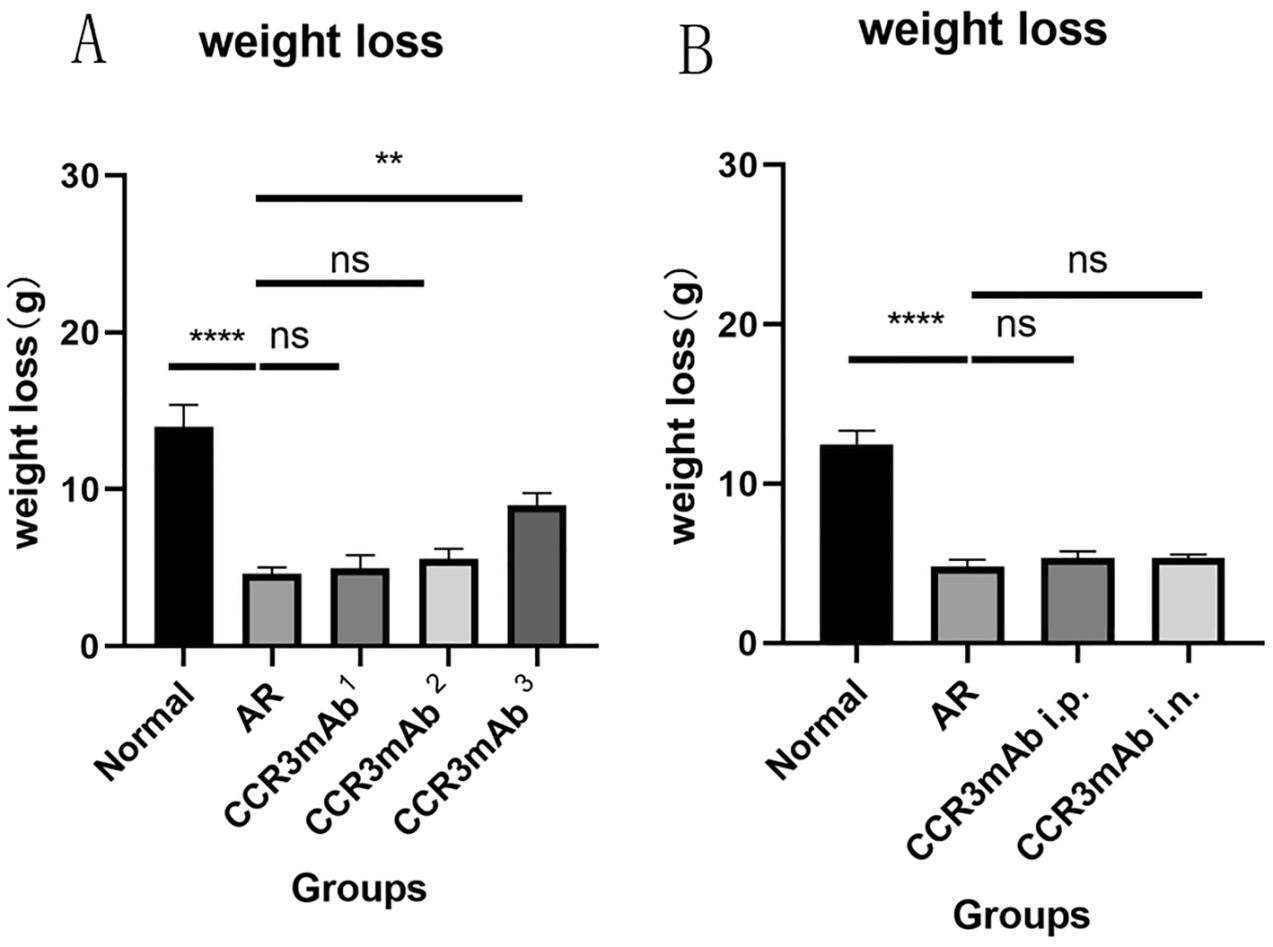
(**A**) Gradient concentration administration: Net body weight increase in mice. 11 (**B**) Different modes of administration: Net body weight gain.

In our study, the net weight gain of experimental mice in the normal control group was significantly greater than that in the allergic rhinitis group and the treatment groups 1 and 2, with no significant difference (P < 0.05), and there was a statistical difference compared with the treatment group 3 (Fig. 11A)^23^. Our results demonstrate that allergic rhinitis can slow down the growth and development of the whole body, and the use of CCR3 monoclonal antibodies can improve this condition of delayed growth and development, and the therapeutic antibody concentration is sufficient to have a significant therapeutic effect when it is effective.

Compared with the allergic group, there was no significant difference in the weight net increase between the CCR3mAb i.p. group and the CCR3mAb nd group (Fig. 11B).

## Discussion

This study aimed to investigate the therapeutic effects of CCR3 monoclonal antibodies on allergic rhinitis in mice. Current research has found high expression of Eotaxin and RANTES in the nasal mucosa and lung tissue of AR models, which is associated with the abundant infiltration of eosinophils (EOS) in AR models. This indicates a significant consistency between the upper and lower respiratory tract inflammation response. Based on the theory of "one airway, one disease", using CCR3 monoclonal antibodies for allergic rhinitis should also have similar effects. Therefore, this experiment used CCR3 antibodies to inhibit the actions of CCR3 and treat allergic rhinitis in mice. The study examined the changes in behavior, pathology (nasal mucosa and lung tissue), and peripheral blood cytokine levels in the model mice^24–27^.

The CCR3 mAb was administered intraperitoneally (i.p.) through the use of an animal model. The clinical manifestations (scratching the nose, sneezing, head breathing, rapid breathing, wheezing, abdominal contractions, restlessness), treatment group 2 and 3 showed significant improvement compared to the control group (AR). HE staining revealed that the inflammatory infiltration in the nasal mucosa of the treatment groups was also significantly improved compared to the AR group, and the histological features of the nasal mucosa were significantly improved. In the lung tissue, only treatment group 3 showed a slight allergic protective effect. This requires further investigation into whether the antibody treatment has a protective effect on lung allergies or if it is a coincidental phenomenon. However, the ELIAS assay showed that the concentration of various cytokines and inflammatory mediators (IFN-γ, IL-2, IL-4, IL-5, IL-13) in the peripheral blood was improved compared to the AR group. Treatment group 2 and 3 showed a significant increase in TH1 cell factors and a decrease in TH2 cell factors compared to the allergen group (P < 0.05). However, there was no significant trend in body weight gain. Overall, the behavioral, pathological and severity analyses of inflammation showed that the order of severity of the condition was: AR > CCR3mAb^1^ > CCR3mAb^2^ > CCR3mAb^3^ > Normal.

It was observed that CCR3 mAb i.p. had more prominent therapeutic effects than nasal administration. However, there was no significant therapeutic effect in treatment group 1, possibly due to insufficient absorption of CCR3 mAb by mice, reduced concentration unable to exert therapeutic effects, or eventually being absorbed into the bloodstream by the abdomen after i.p. administration, thus exerting inhibitory actions against CCR3. In this study, CCR3 mAb was used to treat allergic rhinitis. Based on these data, it was initially discovered that CCR3 mAb can control the clinical symptoms of allergic rhinitis and can affect the pathogenesis of AR by inhibiting inflammatory factors.

In terms of dosing comparison experiments, it was observed that CCR3 mAb i.p. had more significant effects than nasal administration in terms of behavior, pathology, and detection of cytokines. There are two possible reasons: firstly, some antibodies are eliminated by cilia clearance after intranasal administration; secondly, the absorption of antibodies via the nasal mucosa and respiratory tract is not sufficient. In addition, the stability of the therapeutic effects of this antibody used for treatment was not stable enough in this experiment, which may also contribute to the difference in therapeutic effects. This study only made a preliminary and meaningful exploration in animal experiments. The role and mechanism of CCR3 mAb in allergic rhinitis need further in-depth research.

Based on previous research in this field, the Eotaxin-CCR3 axis is an important pathway. Under inflammatory conditions, EOS differentiates and matures through Eotaxin-CCR3 recruitment to the inflammatory site. Therefore, CCR3 may be a target gene for the study of respiratory allergic diseases. Monoclonal antibodies (mAb) are one of the most important types of biological agents after vaccines and recombinant proteins, which have been successfully applied to the treatment of autoimmune diseases, tumors, and allergic diseases. All results demonstrate that the use of CCR3mAb has a significant relieving effect on respiratory allergy in mice. The mechanism is that CCR3mAb binds to the Fc domain of CCR3 expressed on the surface of mast cells, eosinophils, and other cells via its crystallizable region (Fc), thus blocking the Eotaxin/CCR3 pathway.Subsequently, it inhibits a series of activities downstream of mast cells and eosinophils (differentiation maturation, chemotaxis recruitment, cytokine and inflammatory mediator release and degranulation), thereby achieving the goal of relieving respiratory allergy.Using CCR3 mAb can block the CCR3-Eotaxin axis to relieve allergic-related diseases. Moreover, CCR3 is the only receptor for Eotaxin on EOS surface, so anti-CCR3 mAb have strong specificity and high potency against eosinophils^28,29^.

In conclusion, using monoclonal antibodies to target inflammatory cells can suppress the development of inflammatory cells during inflammation processes. This experiment has far-reaching significance for future development of monoclonal antibody drugs and treatment of allergic diseases.

## Materials and methods

### Animals

The experimental animals were 6–8 weeks old Female BALB/c healthy mice, with a body weight of 25–35 g, purchased from the Department of Animal Science, Nanchang University Medical College, Nanchang, Jiangxi Province. All animals were housed in the standard animal room of Nanchang University Laboratory and maintained at a constant temperature of 20℃ with standard mouse feed and high-temperature sterilized drinking water. All procedures were approved by the Welfare Ethics Committee of Nanchang University. All experiments were performed by relevant named guidelines, regulations, and the ARRIVE guidelines.

### Experimental method

#### Experiment one: concentration experiment

Balb/c mice were divided into five groups: (i) Normal mice group (Normal), (ii) Allergic rhinitis group (AR), (iii) Intraperitoneal injection of CCR3 monoclonal antibody CCR3mAb^1^ (5 mg/kg), (iv) Intraperitoneal injection of CCR3 monoclonal antibody CCR3mAb^2^ (10 mg/kg), (v) Intraperitoneal injection of CCR3 monoclonal antibody CCR3mAb^3^ (20 mg/kg), 5 white mice in each group, total 25 mice.

##### Establishment of allergic rhinitis animal model

Female BALB/c mice aged 6–8 weeks were intraperitoneally injected with 200 ul (100 ug OVA and 4 mg AL(OH)3) on days 1, 8, and 15 to establish a basic allergy model. On days 20–26, the challenge solution containing 20 ul (600 ug OVA) was dripped into each nostril twice a day to establish an allergic rhinitis mouse model^30,31^. Mice were anesthetized with isoflurane (1–5%) for induction and maintenance of anesthesia for 24 h after the last stimulation. The mice were subsequently executed and the material was taken.

##### CCR3 mAb interference

On days 24–26 (interval of 1 day, twice a day), mice in groups III, IV, and V were intraperitoneally injected with 5, 10, and 20 mg/kg mouse anti-mouse CCR3 monoclonal antibody dissolved in PBS solution twice a day (left and right injected separately). The interference was carried out 30 min before the challenge.

#### Experiment two: dosage form experiment

##### Experimental method

Balb/c mice were divided into four groups: (i) Normal mice group (Normal), (ii) Allergic rhinitis group (AR), (iii) Intraperitoneal injection of CCR3 monoclonal antibody CCR3mAb2 (10 mg/kg), (iv) Nasal drip of CCR3 monoclonal antibody CCR3mAb3 (20 mg/kg), 5 white mice in each group, total 20 mice.

##### Establishment of an animal model of allergic rhinitis

The 6–8 weeks male BALB/c mice were used on day 1, 8 and 15 days by intraperitoneal injection of sensitizing solution 200 ul (100 ugOVA and 4 mg AL(0H)3), once a day, basic sensitization was performed, and on the 20th–26th day, 20 ul (600 ugOVA) of excitation solution, 10ul/side nasal drip excitation was performed twice a day, to construct the mouse model of allergic rhinitis. Mice were anesthetized with isoflurane (1–5%) for induction and maintenance of anesthesia for 24 h after the last stimulation. The mice were subsequently executed and the material was taken.

##### CCR3 mAb interference

CCR3 mAb was interfered on days 24–26 (1 day interval, 2 times/day) as follows:

1. Intraperitoneal injection: Group (iii) mice were given 10 mg/kg murine anti-mouse CCR3 mAb dissolved in PBS solution for intraperitoneal injection twice a day (left and right injections), and the interference was carried out 30 min before excitation.
2. Nasal drip: Group (iv) mice were given 20 mg/kg mouse anti-mouse CCR3 mAb dissolved in PBS solution for nasal drip interference twice a day at an interval of not less than 8 h.

### Quantitative analysis of eosinophils in nasal mucosa

After removing the nasal mucosa from mice, it was stained with HE and scanned under a microscope. The number of eosinophils was counted in at least 5 randomly selected areas under the X400 lens on each slide.

### Measurement of nasal mucosal thickness

Mice were anesthetized and their nasal mucosa was marked and stored in a − 80 °C freezer for later use. After retrieving the nasal mucosa, it was stained with HE and scanned under a microscope. The thickness of the nasal mucosa was measured in at least 5 randomly selected areas under the X400 lens on each slide.

### Levels of IFN-γ, IL-2, IL-4, IL-5, and IL-13 in peripheral blood

After mouse death, peripheral blood was collected and incubated at 4 °C for 1 day before centrifuging at 3000 rpm for 10 min. The upper serum was used for cytokine detection. Cytokine levels of IFN-γ, IL-2, IL-4, IL-5, and IL-13 in the serum were detected using an immune analytical kit according to the manufacturer's instructions, by enzyme-linked immunosorbent assay (ELISA).

### Net body weight gain

Weight changes in mice are difficult to quantify, so the difference in body weight gain was measured. Mice were weighed and the net body weight gain between days 0 and day 27 before being killed was recorded as the result.

### Data analysis

Statistical software (Graph Pad Prism, San Diego, CA, USA) was used to perform t-test and Mann–Whitney test on the results from different groups. P-values < 0.05 were considered statistically significant. All results are represented as mean ± standard error of the mean (SEM).

## Data Availability

All data generated or analyzed during this study are included in this article (and its Supplementary Information files).
